# Antibodies to *Plasmodium falciparum* merozoite surface protein-1p19 malaria vaccine candidate induce antibody-dependent respiratory burst in human neutrophils

**DOI:** 10.1186/s12936-015-0935-5

**Published:** 2015-10-15

**Authors:** Charlotte Joos, Marie-Louise Varela, Babacar Mbengue, Annick Mansourou, Laurence Marrama, Cheikh Sokhna, Adama Tall, Jean-François Trape, Aissatou Touré, Odile Mercereau-Puijalon, Ronald Perraut

**Affiliations:** Unité d’Immunologie, Institut Pasteur de Dakar, Dakar, Senegal; Unité d’Immunologie Moléculaire des Parasites, Institut Pasteur, Paris, France; Unité d’Immunogénétique/UCAD, Institut Pasteur de Dakar, Dakar, Senegal; Unité d’Epidémiologie, Institut Pasteur de Dakar, Dakar, Senegal; Institut de Recherche pour le Développement (IRD), URMITE, Dakar, Senegal

**Keywords:** Malaria, *Plasmodium falciparum*, ELISA, IgG, Functional assay, Chemiluminescence, Neutrophils, ADRB, MSP1p19, Senegal

## Abstract

**Background:**

Identification of plasmodial antigens targeted by protective immune mechanisms is important for malaria vaccine development. Among functional assays, the neutrophil antibody-dependent respiratory burst (ADRB) induced by opsonized *Plasmodium falciparum* merozoites has been correlated with acquired immunity to clinical malaria in endemic areas, but the target merozoite antigens are unknown. Here, the contribution of antibodies to the conserved C-terminal domain of the *P. falciparum* merozoite surface protein-1 (PfMSP1p19) in mediating ADRB was investigated in sera from individuals living in two Senegalese villages with differing malaria endemicity.

**Methods:**

Anti-PfMSP1p19 antibody levels in sera from 233 villagers were investigated and the involvement of anti-PfMSP1p19 antibodies in ADRB was explored in a subset of samples using (1) isogenic *P. falciparum* parasite clones expressing *P. falciparum* or *Plasmodium chabaudi* MSP1p19; (2) PfMSP1p19-coated plaque ADRB; and, (3) ADRB triggering using sera depleted from PfMSP1p19 antibodies by absorption onto the baculovirus recombinant antigen.

**Results:**

ADRB activity correlated with anti-PfMSP1p19 IgG levels (P < 10^−3^). A substantial contribution of PfMSP1p19 antibody responses to ADRB was confirmed (P < 10^−4^) in an age-adjusted linear regression model. PfMSP1p19 antibodies accounted for 33.1 % (range 7–54 %) and 33.2 % (range 0–70 %) of ADRB activity evaluated using isogenic merozoites (P < 10^−3^) and depleted sera (P = 0.0017), respectively. Coating of PfMSP1p19 on plates induced strong ADRB in anti-PfMSP1p19-positive sera.

**Conclusion:**

These data show that naturally acquired *P. falciparum* MSP1p19 antibodies are potent inducers of neutrophil ADRB and support the development of PfMSP1p19-based malaria vaccine using ADRB assay as a functional surrogate for protection.

**Electronic supplementary material:**

The online version of this article (doi:10.1186/s12936-015-0935-5) contains supplementary material, which is available to authorized users.

## Background

*Plasmodium* spp. parasites responsible for malaria remain a major global health burden and efforts are being intensified to develop effective vaccines and new therapy. *Plasmodium falciparum,* which causes about 600,000 deaths each year [1], is becoming multi-drug resistant [2, 3], exacerbating the need for an effective malaria vaccine. Merozoite surface proteins (MSPs) are attractive candidate antigens for vaccine development and several current vaccine candidates are recombinant MSP analogues [4, 5]. MSPs are expressed by mature intrahepatic forms and as such, are possible targets of cellular effectors. MSPs displayed onto the surface of invasive merozoites are directly accessible to host immune effectors in the blood, such as antibodies, complement, neutrophils, or monocytes [6]. Antibodies against various recombinant MSPs have been associated with protection against clinical episodes of *P. falciparum* malaria in endemic settings [7–9]. The exact function of such antibodies is still poorly understood. Merozoite-specific neutralizing antibodies preventing invasion of red blood cells in human sera were evidenced using invasion assays or growth inhibition assays (GIA), but no clear correlation with protection against malaria morbidity has been documented [9, 10].

There is an increasing body of evidence to associate protection with presence of cytophilic antibodies [11–13]. Antibody-dependent cellular inhibition (ADCI), whereby monocytes activated by antibody-coated merozoites inhibit development of intracellular erythocytic stages has been associated with protection in humans [14]. Salmon et al. [15] and Kumaratilake et al. [16] showed that merozoite-specific antibodies can induce respiratory bursts from neutrophils (ADRB). The mechanism(s) by which antibody triggered ADRB was then investigated in more detail using recombinant antibodies by Pleass et al., who demonstrated the implication of cytophilic antibodies in ADRB [17, 18]. The respiratory burst activity of human polymorphonuclear neutrophils (PMN) triggered by *P. falciparum* merozoites and immune IgG from individuals living in endemic areas has been previously characterized and quantified [19]. Importantly, the antibody-dependent respiratory burst (ADRB) activity significantly correlated with acquired clinical protection, suggesting that the release of extracellular oxygen radicals by activated PMNs may represent a key effector mechanism of naturally acquired immunity to malaria [19].

The identification of the merozoite surface antigens reacting with opsonizing antibodies and with antibodies triggering neutrophil activation is of obvious interest for vaccine development as recently investigated in a *Plasmodium yoelii* malaria mouse model [20].

Here, the role of human antibodies recognizing the C-terminal domain of MSP1, PfMSP1p19, in mediating neutrophil ADRB was investigated. Firstly, the correlation between presence of antibodies to the baculovirus-expressed PfMSP1p19 and ADRB-inducing capacity in the sera from people living in endemic areas was analysed. Secondly, the functional contribution of PfMSP1p19-specific antibodies to ADRB was investigated using *P. falciparum* D10 wild type or transgenic D10 merozoites, expressing the *Plasmodium chabaudi* MSP1p19 orthologue [21]. A direct role for PfMSP1p19-specific antibodies in ADRB was further assessed using sera depleted from specific antibodies by affinity chromatography and solid phase ADRB using PfMSP1p19-coated plates [20, 22]. Results show that PfMSP1p19-specific antibodies account for a good proportion of ADRB activity, providing further support for the development of malaria vaccines including the PfMSP1p19 antigen.

## Methods

### Study sites, subjects and ethics statement

This study is part of a longitudinal study conducted in Dielmo and Ndiop, two Senegalese villages with perennial and seasonal transmission, respectively. The sites, population endemicity and the longitudinal surveys carried out have been described previously [23, 24]. In July 2002, 119 Dielmo and 114 Ndiop villagers were enrolled in a longitudinal and cross-sectional study. At the time of recruitment, no villagers were symptomatic for malaria. The mean age of the Ndiop and Dielmo cohorts was 25.3 years (range 3.4–80.5) and 21.8 years (range 3.9-76.9), respectively; the distribution in the different age groups is shown in Table 1. Blood samples were collected by venous puncture, and sera were stored at −20 °C.

**Table 1:** Antibody responses against MSP1p19 in Dielmo and Ndiop villagers tested for ADRB

Age groups (years)	N	Mean age (years)	Anti-PfMSP1p19 levels^a^	% Positive responders^b^	% High responders^b^	ADRB^c^
DIELMO						
0–6	19	4.6	3.6 [1–9.1]	68	16	154 [46–354]
7–14	31	10.9	3.5 [1–15]	48	13	231 [40–958]
≥15	69	37.4	9.2 [1–16.9]	87	62	380 [106–575]
All	119	25.3	6.8 [1–16.9]	74	42	305 [40–958]
NDIOP						
0–14	49	8.8	6.4 [1–18.4]	80	37	162 [51–545]
15–29	33	20.5	9.2 [1–19.7]	88	55	307 [113–1721]
≥30	32	43.9	11.9 [1–19.9]	94	69	370 [56–1147]
All	114	21.8	8.8 [1–19.9]	86	51	262 [51–1721]

^a^Mean [range] antibody levels to PfMSP1p19 expressed in OD-ratio

^b^Percent of responders (OD-ratio > 2) and of High responders (OD-ratio > 7)

^c^Mean level and range [min–max] of ADRB measured in villagers’ sera

The project protocol and objectives were carefully explained to the assembled villagers, and informed consent, annually renewed, was obtained individually from all subjects either by signature or by thumbprint on a voluntary consent form written in both French and in the local language (Wolof and Serere) [24]. This study was examined and approved by the Senegalese National Health Research Ethics Committee.

Urban samples were from hospitalized adults with confirmed severe malaria, living in the unstable hypo-endemic urban area of Dakar. They were treated at Hôpital Principal, Dakar. Samples were collected day 0 of hospitalization after their use for routine biological investigations. This study was approved by *ad hoc* Ethics Committee and informed consent was obtained from all participants.

### Antigens and antibodies

The soluble recombinant protein corresponding to PfMSP1p19 was produced in the baculovirus/insect cell expression system in High Five (Invitrogen) insect cells. The construct has a C-terminal hexa-histidine tag that replaces the GPI-modification signal sequence of the parasite protein. Recombinant PfMSP1p19 was purified by metallo-affinity chromatography, as described previously [25].

A hyper-immune serum pool (HIS) from 30 immune, primarily adult residents of Dielmo (mean age 36 years, range 9–73 years), and non-immune serum pool (NIS) obtained commercially (Calbiotech, France), were the positive and negative controls, respectively.

### ELISA analysis

The ELISA protocol used to measure PfMSP1p19 antibodies at a 1:200 serum dilution was essentially as described [9, 26, 27], using baculovirus PfMSP1p19 coated on Immulon-4 plates (Dynatech) at 0.5 µg mL^−1^. For inter-assay comparisons, results were expressed as OD-ratios corresponding to OD-sample/OD-naïve. Positive responders (PR) were individuals with an OD-ratio over 2, corresponding to the mean OD of naïve controls + 2SD. High responders (HR) were individuals with an OD-ratio >7, i.e. the threshold level previously shown to be significantly associated with anti-parasite activity in re-infection study in Ndiop [26].

To monitor specific antibody depletions quantitatively, samples were analysed at 1:200, 1:400 and 1:800 dilutions, and an arbitrary titre was extrapolated using a four-parameter logistic fit from a standardized positive control regression curve on each plate, determined using the HIS pool and serial twofold dilutions starting at 1:200 [28, 29].

### Serum depletion

Sera from high responder individuals to PfMSP1p19 were selected for the depletion studies. Each serum (100 µL) was diluted 1:3 in PBS and incubated with 50 µg of recombinant hexa-histidine tagged PfMSP1p19 protein for 30 min at RT to allow antigen–antibody binding. Packed TALON Metal Affinity Resin (Ozyme) pre-equilibrated with PBS (200 µL), was added and incubated with gentle mixing for 3 h at room temperature (RT), to allow antigen–antibody complex binding via the C-terminal hexa-histidine tag. Depleted sera were recovered in the supernatant after centrifugation without further dilution, so that initial and depleted sera were directly comparable. Effective depletion was checked by ELISA.

### Parasite culture and merozoite preparation

*Plasmodium falciparum* parasites (PAM, an FCR3-like background) and *P. falciparum* D10 (D10-PfM3′) or transgenic D10 merozoites, in which PfMSP1p19 is replaced by the non-cross reactive *Plasmodium chabaudi* orthologue, PcMSP1p19 (also called PcMEGF) [21, 30] were maintained in continuous culture on O^+^ erythrocytes in RPMI supplemented with 0.5 % Albumax and 1 μg mL^−1^ gentamycin, in candle jars [31]. Merozoites were collected as described previously [19] from cultures with greater than 5 % parasitaemia after centrifugation 5 min at 400×*g*, to remove red blood cells (RBCs), followed by a second centrifugation of the supernatant for 20 min at 1500×*g*.

### PMN preparation

PMNs were prepared as described previously [19]. Briefly, blood samples from six to seven healthy donors were collected into EDTA-K3 tubes, layered onto Ficoll-Histopaque (density 1.077, Sigma) and centrifuged at RT for 30 min at 400**×***g*. PMNs were harvested at the Ficoll-RBC interface and residual RBCs were lysed by incubation in 8.32 g L^−1^ NH_4_Cl, 0.8 g L^−1^ sodium bicarbonate, and 0.043 g L^−1^ EDTA for 8 min at 4 °C. PMNs were washed twice with Hank’s balanced salt solution (HBSS), enumerated using Trypan blue, and resuspended in PBS at 1–5 × 10^7^ cells mL^−1^.

### Chemiluminescence monitoring and determination of standardized ADRB index

Chemiluminescence was measured as described previously [19] using opaque 96-well plates (Berthold), and a MicroLumat Plus 96 luminometer (Berthold). Briefly, merozoite pellets (40 µL) were incubated with 10 µL of test or control sera for at least 30 min at 37 °C. PMN (100 µL at 1–5 × 10^7^ cells mL^−1^) and isoluminol (100 µL of 1:100 dilution in PBS of 4 mg mL^−1^ stock in DMSO) were loaded rapidly using an Eppendorf multipipette 4780. To facilitate rapid handling, only 40–50 wells per plate were used using the HIS as systematic internal control in the first and last wells. Plate reading started immediately, and continued for 1 h.

Data are presented as standardized activity index of merozoite ADRB calculated as

$${\text{ADRB index}} = \, \left( {{\text{rlu maximum sample}}/{\text{rlu maximum HIS}}} \right) \, \times 1000$$

where rlu maximum HIS is an average of the first and last wells on the plate. Only experiments in which the rlu maximum HIS was ≥100 (≥6 × background), were included in the analyses. An additional internal control with the same positive serum was included in each run.

### Chemiluminescence assay using antigen coated on plates

Baculovirus PfMSP1p19 was coated on white Nunc opaque Maxisorp plates (Dynatech) at 1 µg/mL overnight at 4 °C. Plates were then washed three times with PBS-Tween-0.05 % and blocked for 1 h with PBS-BSA2 % before a second wash. Native (undecomplemented) sera diluted 1:5 in PBS were then added and incubated for 1 h at 37 °C. PMN and isoluminol were then added as for classical ADRB (see above) [19, 22], before reading in the MicroLumat Plus 96 luminometer (Berthold).

### Statistical analysis of ADRB assay data

ELISA and/or ADRB data were analysed for statistical significance using the Wilcoxon signed rank test and the Spearman rank correlation test for non-normally distributed data, and *P* values <0.05 were considered significant. Multiple regression analysis including age of individuals, Ab response to PfMSP1p19 and ADRB was done using R software.

## Results

### Prevalence and levels of PfMSP1p19-binding IgG and their relationship with antibody-dependent respiratory burst activity in individual sera

The prevalence and levels of IgG specific for PfMSP1p19 were first determined in a set of 233 endemic sera, including 119 from Dielmo (holo-endemic) and 114 from Ndiop (meso-endemic). Antibody responses expressed as ELISA OD ratios are summarized in Table 1. Prevalence of IgG to PfMSP1p19 was high, with 74 and 86 % of responders in the Dielmo and Ndiop cohorts, respectively. Both seropositivity and IgG levels to PfMSP1p19 were higher in Dielmo than Ndiop (*P* = 0.023 and *P* = 0.009 by Fisher’s exact test and Mann Withney rank sum test, respectively).

**Fig. 1:**
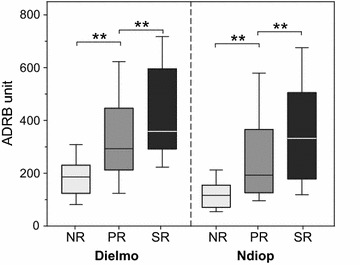
ADRB responses stratified by anti PfMSP1p19 antibody levels in the two villages. ADRB measured in 119 and 114 sera from Dielmo and Ndiop, respectively are plotted as function of their anti-PfMSP1p19 antibodies responses stratified into three levels: non-responders (NR), positive responders (PR, OD ratio > 2) and strong responders (SR, OD ratio > 7). The SR level was selected according to previous study [21] underlining this critical threshold of antibody levels to PfMSP1p19 in Ndiop. *Boxplot* graphs show that the profile of ADRB responses strongly and significantly increase (P < 10^−3^) with increasing anti-PfMSP1p19 antibodies content. The median is indicated by a *line*, the 50 % percentiles indicated by the *box limits* and the *upper* and *lower* 25 % by *whiskers*

ADRB and antibodies to PfMSP1p19 correlated with age. A quite significant correlation between the magnitude of ADRB index and IgG responses to PfMSP1p19 was found (Rho = 0.71 and 0.68, *P* < 10^−3^ for Dielmo and Ndiop, respectively). Stratification of antibody responses to PfMSP1p19 of individuals (Fig. 1) into non-responders (NR), positive responders (PR, individuals with OD-ratio >2) and high responders (HR; individuals with OD-ratio >7) outlined a significant association of ADRB with increasing response to PfMSP1p19 (*P* < 10^−3^). The significant contribution of PfMSP1p19 antibody responses to ADRB was confirmed highly significant *P* < 10^−4^ in an age-adjusted linear regression model.

### Naturally acquired antibodies binding to PfMSP1p19 are major inducers of ADRB activity

**Fig. 2:**
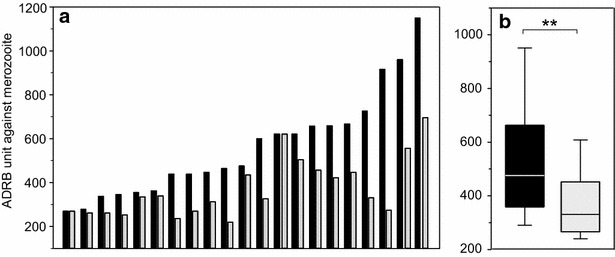
Naturally-acquired anti-PfMSP1p19 antibodies are major inducers of ADRB activity against *P. falciparum* merozoites. **a** ADRB chemiluminescence readout for 21 sera with high anti-PfMSP1p19 OD-ratios tested using either *P. falciparum* D10 merozoites (D10-PfM3′, *black bars*) or transgenic D10 merozoites in which the *P. falciparum* MSP1p19 gene was replaced by its *P. chabaudi* orthologue (D10-PcMEGF, *black bars*). D10-PfM3′ and D10-PcMEGF merozoites were tested in the same plate, with the same polymorphonuclear cells batch. **b** *Boxplot* graph showing the significant (P < 10^−3^) overall distribution of ADRB generated using sera and D10-PfM3′ or D10-PcMEGF transgenic merozoites. The median is indicated by a *line*, the 50 % percentiles indicated by the *box limits* and the *upper* and *lower* 25 % by *whiskers*

To demonstrate a role for PfMSP1p19-specific IgG in the ADRB, assays were carried out using either *P. falciparum* D10 (D10-PfM3′) or transgenic D10 PcMEGF merozoites, in which PfMSP1p19 has been replaced by the non-cross reactive *P. chabaudi* homologue, PcMSP1p19 [30], such that differences in observed chemiluminescence signals can be attributed to antibodies specific for PfMSP1p19 [21]. As shown in Fig. 2, comparative ADRBs were performed using 21 individual sera with high anti-PfMSP1p19 ELISA OD-ratios. Chemiluminescence elicited by the transgenic PcMEGF merozoites was significantly reduced compared to D10-PfM3′ for all individual sera (*P* < 10^−3^), varying from 7 to 54 % inhibition, with a mean of 33.1 %. Importantly a similar reduction of chemiluminescence was observed using the HIS pool of 30 immune antisera, none of which was selected for presence of PfMSP1p19-binding antibodies (and thus unrelated to the 21 individually sera analysed above). These results indicate that antibodies induced by natural *P. falciparum* infection to this single conserved antigen represent a good proportion of antibodies mediating reactive oxygen species (ROS) release by neutrophils.

### Impact of PfMSP1p19 antibody depletion on ADRB activity

To explore contribution of PfMSP1p19-specific antibody to the ADRB readout, another approach was developed, involving depletion of specific antibodies by adsorption on the recombinant antigen. Efficiency of depletion monitored by ELISA on the antigen showed that the depletion protocol was highly effective. Median titre units decreased from 511 to 1 after anti-PfMSP1p19 depletion, indicating a depletion efficiency >99 %.

**Fig. 3:**
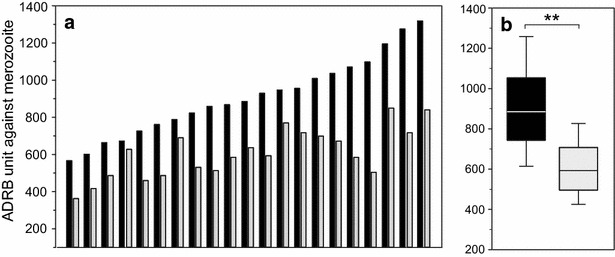
Effect of anti-PfMSP1p19 depletion on ADRB activity against *P. falciparum* merozoites. A set of 21 sera were depleted from anti-PfMSP1p19 antibodies using metal affinity immuno-adsorbent. **a** The ADRB level of each serum pair (undepleted and depleted, *black* and *light bars*, respectively) was plotted in increasing order of ADRB level of undepleted sera. **b** *Boxplot* graph showing the significant (P < 10^−3^) overall drop of ADRB from initial vs depleted sera. All sera selected had initial high levels of anti-PfMSP1p19 antibodies. The median is indicated by a *line*, the 50 % percentiles indicated by the *box limits* and the *upper* and *lower* 25 % by *whiskers*

Figure 3 summarizes the effect of anti-PfMSP1p19 antibody depletion on ADRB activity. A paired comparison of ADRB indexes before and after depletion of anti-PfMSP1p19 in 21 individual immune sera showed a 33.2 % average reduction after depletion (range 0–70 %; *P* = 0.0017), confirming the results obtained using the transgenic D10 PcMEGF merozoites.

### Solid-phase ADRB driven by PfMSP1p19 antigen and human sera

To further explore the contribution of PfMSP1p19-specific antibody to ADRB, solid-phase ADRB was carried out using plates coated with the recombinant PfMSP1p19 antigen as sole antigen in the assay. A set of 21 sera from Dielmo and Ndiop and four sera from hospitalized urban malaria with variable levels of anti-PfMSP1p19 IgG were simultaneously tested.

The kinetics of luminescence and ROS production was different than from the previously described ADRB assay using entire merozoites [19]. The peak of luminescence, measured using isoluminol occurred around 30 min, i.e. later than the peak occurring within 5 min in the usual the standard assay (Additional file 1) [19, 22]. High levels of luminescence were observed (around 2–3000 rlu), the highest signal being produced by the immune IgG positive control. Background rlu from the commercial naive control pool of sera was around 300 and very low signals were observed with individual naive European sera (rlu < 40).

**Fig. 4:**
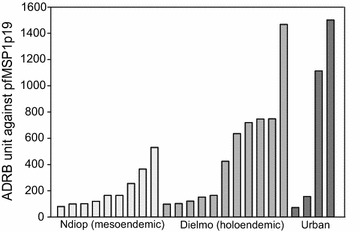
ADRB activity of a set of 24 sera in the *solid-phase* assay using PfMSP1p19-coated plates. Histograms of ADRB activity measured using PfMSP1p19-coated in 9, 11 and 4 sera from Ndiop (*light grey*), Dielmo (*medium grey*) and urban malaria (*dark grey*), respectively, plotted in increasing order of ADRB units, respectively. The sera were chosen irrespective of their level of antibodies to PfMSP1p19 antigen

The individual levels of ADRB measured in the set of sera are shown Fig. 4. The solid-phase PfMSP1p19-specific ADRB index varied depending on the individual. Importantly, there was a positive and significant relationship between IgG to PfMSP1p19 and solid-phase ADRB measures, with a correlation coefficient rho = 0.64 (*P* < 10^−3^).

## Discussion

It has been previously demonstrated that PMN from healthy donors produce a respiratory burst in response to merozoites and IgG1 and/or IgG3 antibodies in sera from individuals living in malaria-endemic areas [19]. It was further shown that the merozoite-triggered ADRB activity of sera from humans living in endemic areas correlated with naturally acquired clinical protection from malaria. The work presented here identifies PfMSP1p19 as a target of antibodies mediating ADRB activity in sera from humans immunized by repeated exposure to *P. falciparum* infection. IgG responses to the recombinant PfMSP1p19 were highly correlated with ADRB activity in the two settings analysed. Importantly, depletion of sera from antibodies specific for PfMSP1p19 or use of merozoites expressing the non-cross-reactive rodent orthologue, PcMSP1p19 caused a significant reduction in the capacity of hyper-immune and high responder sera to mediate ADRB activity. Finally, ADRB could be triggered by incubating immune sera with recombinant PfMSP1p19 as sole target antigen.

The clinical implications of this work are two-fold. First, the results presented here strongly implicate human IgG antibodies’ ability to react with the native PfMSP1p19 antigen displayed onto the merozoite surface as mediators of ADRB activity. Importantly, this target antigen exhibits limited antigenic diversity, an essential feature of vaccine candidates aiming to provide durable protection against polymorphic field parasite populations. Numerous sero-epidemiology studies have documented a high seroprevalence of antibodies to PfSMP1p19 [8, 9, 27, 32, 33]. The data observed here with two Senegalese cohorts living in different endemic conditions and moreover gathering different ethnic groups [34] are thus likely to be relevant for multiple endemic settings. Natural antibodies to PfMSP1p19 accounted for approximately 30 % of the ADRB activity of immune human sera in this work. Interestingly, O’Donnell et al. [30] using immune sera from Papua New Guinea and the same isogenic parasite strains showed that antibodies to PfMSP1p19 were responsible for about 25 % of the erythrocyte invasion inhibitory response of the human sera in vitro [30]. Thus these two assays provide a similar evaluation of the contribution of the anti-PfMSP1p19 specificity within the pool of antibodies reacting with merozoites. This indicates that indeed antibodies to PfMSP1p19 are a major component of the naturally-acquired, infection-elicited anti-merozoite response. A recent study conducted in the *P. yoelii* mouse model showed that vaccination with the AdHu5-PyMSP1_42_ construct did not induce anti-PyMSP1p19 ADRB-triggering antibodies. Anti-PyMSP1p19 antibodies contributing to ADRB were however observed in naïve as well as vaccinated mice after a primary infection, suggesting that infection elicits such antibodies [20].

Second, the data show that the baculovirus recombinant PfMSP1p19 recapitulates here the functional triggering activity of the merozoite-displayed native antigen. This is important information for vaccine development. *Plasmodium* MSP1p19 is composed of two intimately associated EGF domains, including 10–12 cysteines and five to six disulfide bonds, constituting an overall conformation not easily reproduced in lower order expression systems [35, 36], and human antibody recognition of PfMSP1p19 is totally conformation dependent [25, 37]. Moreover, evidence from the literature points to the potential importance of proper MSP1p19 conformation in evaluating the effectiveness of specific antibody responses. Anti-baculovirus PfMSP1p19 IgG levels in endemic sera from Dielmo and Ndiop were previously shown to correlate with both erythrocyte invasion inhibition by *P. falciparum* merozoites in vitro, and clinical protection from *P. falciparum* malaria in an age-adjusted multivariate analysis [9] (confirmed in two subsequent studies, R. Perraut and ML Varela, unpublished data). These results contrast with those of Roussilhon et al. [13] using sera from the same Senegalese sites, indicating no correlation of clinical protection with antibodies to a recombinant PfMSP1-19 construct corresponding to a single EGF domain with unlikely resemblance to native PfMSP1p19 [35, 36]. Similarly Dodoo et al. [32] found that IgG1 specific for baculovirus PfMSP1p19 was correlated with protection from clinical malaria in Ghanaian children, whereas a previous study in the same location using *Escherichia coli* GST-PfMSP1p19 fusion antigens showed no such association [38].

In the work reported here, focus was on IgG. However, ADRB was measured with immune sera, which might also contain specific IgA. The contribution of IgA to the observed responses remains to be investigated, as IgA has been shown to induce respiratory bursts to merozoites and MSP1-19 via FcαR (CD89), which is constitutively expressed by neutrophils [18].

The association of antibodies to PfMSP1p19 with protection is incompletely captured using parameters such as antibody levels [7, 9, 13, 26, 27, 32, 38, 39] or GIA [8, 9]. The association with ADRB, itself associated with protection against clinical malaria, may somehow combine previous observations: high levels of anti-PfMSP1p19 antibodies that recognize the merozoite surface-displayed antigen may altogether inhibit invasion, efficiently trigger ADRB and possibly ADCI. These functional read-outs are not mutually exclusive, reflecting an association of antibodies with the native antigen on the merozoite surface. ADRB is rapid and as such stands out as a convenient assay for monitoring the anti-merozoite activity in human sera, and an interesting in vitro surrogate of clinical protection relevant for MSP-based vaccine candidates.

Here, three experimental approaches were used to study the contribution of specific antibodies to ADRB. Use of isogenic merozoites was appropriate for MSP1p19, because its function is apparently based entirely on its 2 EGF-domain structure, and can be brought about by an orthologous domain with differing antigenicity. This feature is unusual and may not apply to other antigens of interest [21]. Moreover, isogenic lines pairs expressing or not a specific antigen, are still rare. The data shown here validate the depletion approach and the solid-phase ADRB approach for the evaluation of other MSP vaccine candidates using the ADRB assay. The depletion protocol depended on using hexa-histidine-tagged antigens, which allowed the formation of antigen–antibody complexes in solution and subsequent removal by binding to the metallo-affinity resin, which was critical for efficient depletion. This methodology should be readily adaptable to antigens carrying other specific tags and presents the advantage of not requiring preparation of specific immuno-affinity chromatography reagents. The antigen-coated ADRB proved a convenient test for functional screening of polyclonal sera for single antigen specificity such as for PfMSP1p19. However, the stimulation is rather qualitative, with a positive but not very strong correlation (65 %) with level of antibodies measured by ELISA. The solid phase ADRB, where PMN activation is detected by the use of isoluminol, i.e., monitoring predominantly extracellular ROS, differs from the rapid peak response (within 5 min) in standard ADRB using merozoites [19]. Kapeslki et al. reported that the peak of luminescence occurred later (20 min) when non-decomplemented plasma are used [22]. However, such a solid-phase test is easier of use than merozoite-based ADRB. Further improvement is required for optimization e.g. using IgG instead of sera, HBSS medium instead of PBS [40], luminol instead of isoluminol [22] and should conveniently complement the process of development for functional analysis targeting merozoite-derived vaccine candidates.

## Conclusions

The results presented here show that antibodies to a single small conserved antigen, PfMSP1p19, are responsible for a good proportion of neutrophil ADRB, an activity that has been correlated with protection from clinical *P. falciparum* malaria in endemic areas. They provide further support for development of baculovirus recombinant PfMSP1p19 as a vaccine candidate. Importantly, the work opens the door to a new approach for the functional analysis of antibodies to other merozoite surface antigens, as a complement to the GIA and ADCI assays.

## Additional file

10.1186/s12936-015-0935-5 Exemple of ADRB luminescence profiles recorded in the study using the standard ADRB assay (A) and the solid-phase PfMSP1p19-coated ADRB assay (B). In (B) the individual luminescence units (rlu) recorded using luminometer as function of time were measured in the reference HIS used as positive control in all assays (a), two PfMSP1p19-positive individuals from Dielmo and Ndiop (b, c), one PfMSP1p19-negative individuals from Dielmo (d), a pool of European sera used as negative control (e) and an individual European negative control (f).

## Abbreviations

ADCI: antibody dependent cellular inhibition
ADRB: antibody dependent respiratory burst
HIS: hyper-immune sera
MSP: merozoite surface protein
PMN: polymorphonuclear neutrophils
rlu: relative light units
ROS: reactive oxygen species
GIA: growth inhibition assay
NIS: non-immune sera
RT: room temperature
NR: non-responders
PR: positive responders
HR: high responders
