# Supplementary figures and images for: Antibodies to Plasmodium falciparum merozoite surface protein-1p19 malaria vaccine candidate induce antibody-dependent respiratory burst in human neutrophils
Source: Malar J. 2015 Oct 15;14:409. doi: 10.1186/s12936-015-0935-5 (PMC4608189; doi:10.1186/s12936-015-0935-5)

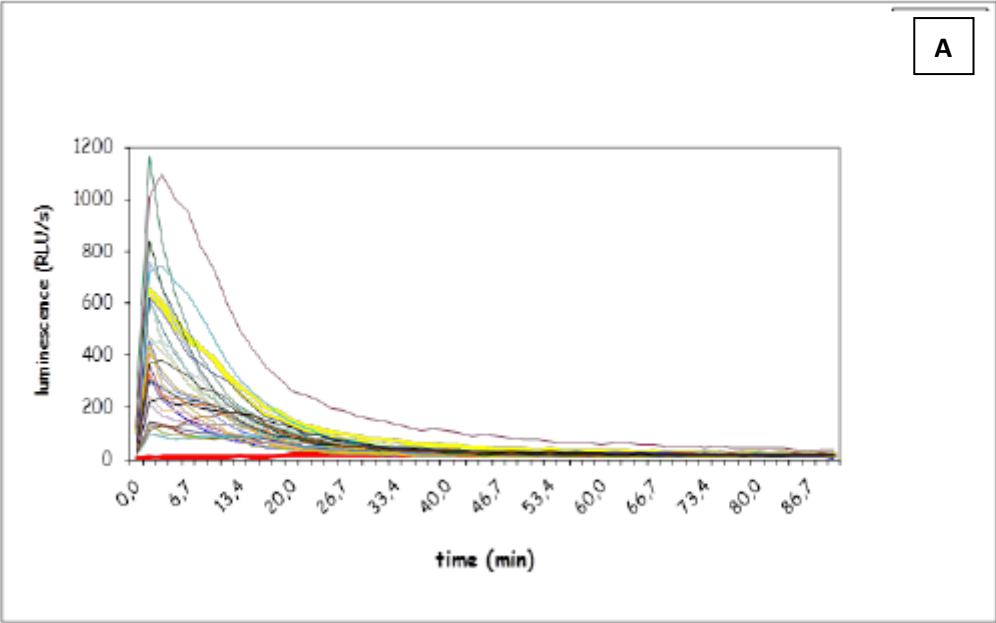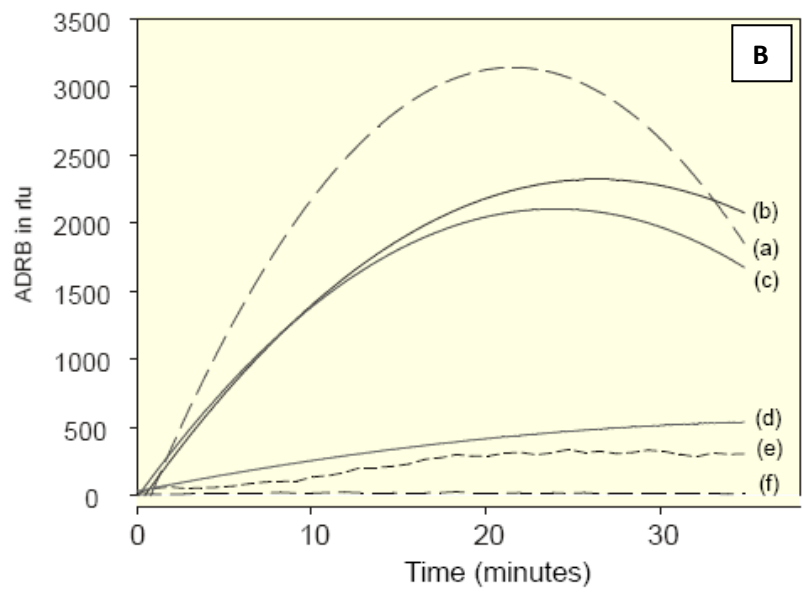

Supplement: Supplementary file 1 — 10.1186/s12936-015-0935-5 Exemple of ADRB luminescence profiles recorded in the study using the standard ADRB assay (A) and the solid-phase PfMSP1p19-coated ADRB assay (B). In (B) the individual luminescence units (rlu) recorded using luminometer as function of time were measured in the reference HIS used as positive control in all assays (a), two PfMSP1p19-positive individuals from Dielmo and Ndiop (b, c), one PfMSP1p19-negative individuals from Dielmo (d), a pool of European sera used as negative control (e) and an individual European negative control (f). [file 12936_2015_935_MOESM1_ESM.pdf]
